# Supplementary material for: Yucatan Miniswine Model of Atrial Fibrillation: Clinical Relevance
Source: PLoS One. 2026 Feb 4;21(2):e0340985. doi: 10.1371/journal.pone.0340985 (PMC12871992; doi:10.1371/journal.pone.0340985)
Supplement: Attachment — Submitted filename: Pouria Response to Reviewers Final 12-16-2025 + Header.docx [file pone.0340985.s003.docx]

**PONE-D-25-22478R1 Yucatan Mini Swine Model of Atrial Fibrillation: Clinical Relevance. Response to Reviewers.**

December 15, 2025

Kamal Sharma PhD

Academic Editor

PLOS ONE

Pone-D-25-222478 Yucatan Mini Swine Model of Atrial Fibrillation: Clinical Relevance

Dear Dr. Kamal Sharma

This is our response letter for our manuscript, Pone-D-25-222478, “Yucatan Mini Swine Model of Atrial Fibrillation: Clinical Relevance.” We thank you for the positive review. By responding to your critiques, we feel the manuscript has been strengthened and we thank you for that. Below are our specific responses to each critique. We have included a marked up copy of our manuscript labeled “Revised Manuscript with Track Changes.” We have also included an unmarked version of your revised paper without tracked changes, labelled “Manuscript”. We would like to thank you all for allowing us to change the corresponding author and extend our responses. The first author, Pouria Mostafizi BA, is now a second lieutenant in the US Army stationed at Fort Benning, GA we have kept him up to date with all the changes made, however his position restricted his ability to correspond with PLOS One.

*We have made no changes in our financial disclosures. As suggested, we have deposited our laboratory protocols in protocols.io submitted.*

*1.Please ensure that your manuscript meets PLOS ONE's style requirements, including those for file naming. The PLOS ONE style templates can be found at*

[*https://journals.plos.org/plosone/s/file?id=wjVg/PLOSOne_formatting_sample_main_body.pdf*](https://nam11.safelinks.protection.outlook.com/?url=https%3A%2F%2Fjournals.plos.org%2Fplosone%2Fs%2Ffile%3Fid%3DwjVg%2FPLOSOne_formatting_sample_main_body.pdf&data=05%7C02%7Cgoldmans%40shc.arizona.edu%7C6b7e618d3dce418f2dca08de16452d22%7C5ee35505eb8e4929937d645df5013288%7C1%7C0%7C638972683568890958%7CUnknown%7CTWFpbGZsb3d8eyJFbXB0eU1hcGkiOnRydWUsIlYiOiIwLjAuMDAwMCIsIlAiOiJXaW4zMiIsIkFOIjoiTWFpbCIsIldUIjoyfQ%3D%3D%7C0%7C%7C%7C&sdata=uxCzNfPGLBfvZSL%2BvQcsBs4JAyrlFMD19VbIbGZIGTY%3D&reserved=0) *and*

*2*.As requested, we have included in our Methods Section (1) methods of sacrifice, (2) methods of anesthesia and/or analgesia, and (3) efforts to alleviate suffering.

3. As requested, we have removed funding information from the text of the manuscript. The funding information you included below is correct.

4. *Thank you for stating the following financial disclosure:*

*“This work was supported by Tech Launch Arizona Asset Development Grant UA17-095, the WARMER Research Foundation, and the Sarver Heart Center, University of Arizona.” "The funders had no role in study design, data collection and analysis, decision to publish, or preparation of the manuscript." Please change the online submission form on your behalf.*

We have added this to financial disclosure statement in the additional information section of the manuscript

5. *We note that your Data Availability Statement is currently as follows: All relevant data are within the manuscript and in Supporting Information files.*

*Please confirm at this time whether or not your submission contains all raw data required to replicate the results of your study. Authors must share the “minimal data set” for their submission. PLOS defines the minimal data set to consist of the data required to replicate all study findings reported in the article, as well as related metadata and methods (*[*https://journals.plos.org/plosone/s/data-availability#loc-minimal-data-set-definition*](https://nam11.safelinks.protection.outlook.com/?url=https%3A%2F%2Fjournals.plos.org%2Fplosone%2Fs%2Fdata-availability%23loc-minimal-data-set-definition&data=05%7C02%7Cgoldmans%40shc.arizona.edu%7C6b7e618d3dce418f2dca08de16452d22%7C5ee35505eb8e4929937d645df5013288%7C1%7C0%7C638972683568956884%7CUnknown%7CTWFpbGZsb3d8eyJFbXB0eU1hcGkiOnRydWUsIlYiOiIwLjAuMDAwMCIsIlAiOiJXaW4zMiIsIkFOIjoiTWFpbCIsIldUIjoyfQ%3D%3D%7C0%7C%7C%7C&sdata=b4YcVrMZIRzhadw9lw%2BjwgLpAqUpGeQS225M5o2wIww%3D&reserved=0)*).*

*Authors do not need to submit their entire data set if only a portion of the data was used in the reported study.*

*If your submission does not contain these data, please either upload them as Supporting Information files or deposit them to a stable, public repository and provide us with the relevant URLs, DOIs, or accession numbers. For a list of recommended repositories, please see* [*https://journals.plos.org/plosone/s/recommended-repositories*](https://nam11.safelinks.protection.outlook.com/?url=https%3A%2F%2Fjournals.plos.org%2Fplosone%2Fs%2Frecommended-repositories&data=05%7C02%7Cgoldmans%40shc.arizona.edu%7C6b7e618d3dce418f2dca08de16452d22%7C5ee35505eb8e4929937d645df5013288%7C1%7C0%7C638972683568986507%7CUnknown%7CTWFpbGZsb3d8eyJFbXB0eU1hcGkiOnRydWUsIlYiOiIwLjAuMDAwMCIsIlAiOiJXaW4zMiIsIkFOIjoiTWFpbCIsIldUIjoyfQ%3D%3D%7C0%7C%7C%7C&sdata=7W0LmyWbnjJooQFiia6TidOMztS%2BSPmO7mRgooRlWXg%3D&reserved=0)*.*

We confirm the data availability statement and have uploaded all raw datasets into a figshare repository 10.25422/azu.data.30471077

6. *When completing the data availability statement of the submission form, you indicated that you will make your data available on acceptance. We strongly recommend all authors decide on a data sharing plan before acceptance, as the process can be lengthy and hold up publication timelines. Please note that, though access restrictions are acceptable now, your entire data will need to be made freely accessible if your manuscript is accepted for publication. This policy applies to all data except where public deposition would breach compliance with the protocol approved by your research ethics board. If you are unable to adhere to our open data policy, please kindly revise your statement to explain your reasoning and we will seek the editor's input on an exemption. Please be assured that, once you have provided your new statement, the assessment of your exemption will not hold up the peer review process.*

We confirm the data availability statement and have uploaded all raw datasets into a figshare repository 10.25422/azu.data.30471077

7. Our Methods Section includes the Ethical Considerations: “All animal work was conducted under the oversight of the University of Arizona Institutional Animal Care and Use Committee (IACUC) and the University of Arizona Animal Care (UAC) veterinary staff.”

*8. We note that Figure 1 in your submission contain copyrighted images. All PLOS content is published under the Creative Commons Attribution License (CC BY 4.0), which means that the manuscript, images, and Supporting Information files will be freely available online, and any third party is permitted to access, download, copy, distribute, and use these materials in any way, even commercially, with proper attribution. For more information, see our copyright guidelines:* [*http://journals.plos.org/plosone/s/licenses-and-copyright*](https://nam11.safelinks.protection.outlook.com/?url=http%3A%2F%2Fjournals.plos.org%2Fplosone%2Fs%2Flicenses-and-copyright&data=05%7C02%7Cgoldmans%40shc.arizona.edu%7C6b7e618d3dce418f2dca08de16452d22%7C5ee35505eb8e4929937d645df5013288%7C1%7C0%7C638972683569016430%7CUnknown%7CTWFpbGZsb3d8eyJFbXB0eU1hcGkiOnRydWUsIlYiOiIwLjAuMDAwMCIsIlAiOiJXaW4zMiIsIkFOIjoiTWFpbCIsIldUIjoyfQ%3D%3D%7C0%7C%7C%7C&sdata=KCLiHY3Bwm44tJxZLNtVQGpDFLonzlvHIJgly%2BBuPuQ%3D&reserved=0)*.*

*We require you to either (1) present written permission from the copyright holder to* *publish these figures specifically under the CC BY 4.0 license, or (2) remove the figures from your submission:*

*1. You may seek permission from the original copyright holder of Figure1  to publish the content specifically under the CC BY 4.0 license.*

*We recommend that you contact the original copyright holder with the Content Permission Form (*[*http://journals.plos.org/plosone/s/file?id=7c09/content-permission-form.pdf*](https://nam11.safelinks.protection.outlook.com/?url=http%3A%2F%2Fjournals.plos.org%2Fplosone%2Fs%2Ffile%3Fid%3D7c09%2Fcontent-permission-form.pdf&data=05%7C02%7Cgoldmans%40shc.arizona.edu%7C6b7e618d3dce418f2dca08de16452d22%7C5ee35505eb8e4929937d645df5013288%7C1%7C0%7C638972683569044667%7CUnknown%7CTWFpbGZsb3d8eyJFbXB0eU1hcGkiOnRydWUsIlYiOiIwLjAuMDAwMCIsIlAiOiJXaW4zMiIsIkFOIjoiTWFpbCIsIldUIjoyfQ%3D%3D%7C0%7C%7C%7C&sdata=zNNhaBADaRlkbHFwyFXs6iLVkGd90X4oOGj%2Fzh51LAo%3D&reserved=0)*) and the following text:*

*“I request permission for the open-access journal PLOS ONE to publish XXX under the Creative Commons Attribution License (CCAL) CC BY 4.0 (*[*http://creativecommons.org/licenses/by/4.0/*](https://nam11.safelinks.protection.outlook.com/?url=http%3A%2F%2Fcreativecommons.org%2Flicenses%2Fby%2F4.0&data=05%7C02%7Cgoldmans%40shc.arizona.edu%7C6b7e618d3dce418f2dca08de16452d22%7C5ee35505eb8e4929937d645df5013288%7C1%7C0%7C638972683569072735%7CUnknown%7CTWFpbGZsb3d8eyJFbXB0eU1hcGkiOnRydWUsIlYiOiIwLjAuMDAwMCIsIlAiOiJXaW4zMiIsIkFOIjoiTWFpbCIsIldUIjoyfQ%3D%3D%7C0%7C%7C%7C&sdata=z2AEct6vmPG9syi1eMqdD083JrS%2BGEuAbuZNJJ9vCGg%3D&reserved=0)*). Please be aware that this license allows unrestricted use and distribution, even commercially, by third parties. Please reply and provide explicit written permission to publish XXX under a CC BY license and complete the attached form.”*

*Please upload the completed Content Permission Form or other proof of granted permissions as an "Other" file with your submission.*

*In the figure caption of the copyrighted figure, please include the following text: “Reprinted from [ref] under a CC BY license, with permission from [name of publisher], original copyright [original copyright year].”*

*2. If you are unable to obtain permission from the original copyright holder to publish these figures under the CC BY 4.0 license or if the copyright holder’s requirements are incompatible with the CC BY 4.0 license, please either i) remove the figure or ii) supply a replacement figure that complies with the CC BY 4.0 license. Please check copyright information on all replacement figures and update the figure caption with source information. If applicable, please specify in the figure caption text when a figure is similar but not identical to the original image and is therefore for illustrative purposes only.*

All new images were taken by our own team of the devices, so we do not need copyright permissions. We have replaced all the copyright images with our own images. (Fig 1). For the BioRender image, we have included the citation for our BioRender license (Fig 2).

9. The references for Figures 1-7 are now cited in the text.

*10. Please include captions for your Supporting Information files at the end of your manuscript, and update any in-text citations to match accordingly. Please see our Supporting Information guidelines for more information:* [*http://journals.plos.org/plosone/s/supporting-information*](https://nam11.safelinks.protection.outlook.com/?url=http%3A%2F%2Ftrack.editorialmanager.com%2FCL0%2Fhttp%3A%252F%252Fjournals.plos.org%252Fplosone%252Fs%252Fsupporting-information%2F1%2F010f0197f94e1492-5ae329c4-4a07-4080-ba3c-f3daec144e45-000000%2FlBZw0tD5W-F3rJ4N35y6GHCVPUH2Zypb_tEkD0ms3Fw%3D219&data=05%7C02%7Cgoldmans%40shc.arizona.edu%7C6b7e618d3dce418f2dca08de16452d22%7C5ee35505eb8e4929937d645df5013288%7C1%7C0%7C638972683569100249%7CUnknown%7CTWFpbGZsb3d8eyJFbXB0eU1hcGkiOnRydWUsIlYiOiIwLjAuMDAwMCIsIlAiOiJXaW4zMiIsIkFOIjoiTWFpbCIsIldUIjoyfQ%3D%3D%7C0%7C%7C%7C&sdata=HcE4MOurOLTpUkR1%2BbzU8T3gVuIzIjBKtYLmg1xkfCk%3D&reserved=0)*.*

We have added captions for all figures.

*11. We note you have not yet provided a protocols.io PDF version of your protocol and/or a protocols.io DOI. When you submit your revision, please provide a PDF version of your protocol as generated by protocols.io (the file will have the protocols.io logo in the upper right corner of the first page) as a Supporting Information file. The filename should be S1_file.pdf, and you should enter “S1 File” into the Description field. Any additional protocols should be numbered S2, S3, and so on. Please also follow the instructions for Supporting Information captions [*[*https://journals.plos.org/plosone/s/supporting-information#loc-captions*](https://nam11.safelinks.protection.outlook.com/?url=https%3A%2F%2Fjournals.plos.org%2Fplosone%2Fs%2Fsupporting-information%23loc-captions&data=05%7C02%7Cgoldmans%40shc.arizona.edu%7C6b7e618d3dce418f2dca08de16452d22%7C5ee35505eb8e4929937d645df5013288%7C1%7C0%7C638972683569129170%7CUnknown%7CTWFpbGZsb3d8eyJFbXB0eU1hcGkiOnRydWUsIlYiOiIwLjAuMDAwMCIsIlAiOiJXaW4zMiIsIkFOIjoiTWFpbCIsIldUIjoyfQ%3D%3D%7C0%7C%7C%7C&sdata=PbmjakVTkkV1%2FAR1IsiY1UdC3hcCHtBCqtrz3R4FnTU%3D&reserved=0)*]. The title in the caption should read: “Step-by-step protocol, also available on protocols.io.”*

*Please assign your protocol a protocols.io DOI, if you have not already done so, and include the following line in the Materials and Methods section of your manuscript: “The protocol described in this peer-reviewed article is published on protocols.io (*[*https://dx.doi.org/10.17504/protocols.io.[...]*](https://nam11.safelinks.protection.outlook.com/?url=https%3A%2F%2Fdx.doi.org%2F10.17504%2Fprotocols.io.%5B...%5D&data=05%7C02%7Cgoldmans%40shc.arizona.edu%7C6b7e618d3dce418f2dca08de16452d22%7C5ee35505eb8e4929937d645df5013288%7C1%7C0%7C638972683569159286%7CUnknown%7CTWFpbGZsb3d8eyJFbXB0eU1hcGkiOnRydWUsIlYiOiIwLjAuMDAwMCIsIlAiOiJXaW4zMiIsIkFOIjoiTWFpbCIsIldUIjoyfQ%3D%3D%7C0%7C%7C%7C&sdata=xBiE0OnjVabQH3t7%2Fvb%2FxOCRUOqxRMZcugt90FWXaAI%3D&reserved=0)*) and is included for printing purposes as S1 File.” You should also supply the DOI in the Protocols.io DOI field of the submission form when you submit your revision.*

*If you have not yet uploaded your protocol to protocols.io, you are invited to use the platform’s protocol entry service [*[*https://www.protocols.io/we-enter-protocols*](https://nam11.safelinks.protection.outlook.com/?url=https%3A%2F%2Fwww.protocols.io%2Fwe-enter-protocols&data=05%7C02%7Cgoldmans%40shc.arizona.edu%7C6b7e618d3dce418f2dca08de16452d22%7C5ee35505eb8e4929937d645df5013288%7C1%7C0%7C638972683569188962%7CUnknown%7CTWFpbGZsb3d8eyJFbXB0eU1hcGkiOnRydWUsIlYiOiIwLjAuMDAwMCIsIlAiOiJXaW4zMiIsIkFOIjoiTWFpbCIsIldUIjoyfQ%3D%3D%7C0%7C%7C%7C&sdata=sZEiW8Bo9ldeR4F7AJrMv6qzWHuNo3%2Fyaq%2B7cbLSZwc%3D&reserved=0)*] for doing so, at no charge. Through this service, the team at protocols.io will enter your protocol for you and format it in a way that takes advantage of the platform’s features. When submitting your protocol to the protocol entry service please include the customer code PLOS2022 in the Note field and indicate that your protocol is associated with a PLOS ONE Lab Protocol Submission. You should also include the title and manuscript number of your PLOS ONE submission.*

We have uploaded standard operating procedures for the study under the S1 file attachment.

*12. If the reviewer comments include a recommendation to cite specific previously published works, please review and evaluate these publications to determine whether they are relevant and should be cited. There is no requirement to cite these works unless the editor has indicated otherwise.*

The reviewers have not made a recommendation to cite specific previously published works

Response to **Reviewers' critiques: :**

**Reviewer's Responses to Questions**

**Comments to the Author**

1. Does the manuscript report a protocol which is of utility to the research community and adds value to the published literature?

Reviewer #1: Yes

Reviewer #2: Yes

2. Has the protocol been described in sufficient detail?

To answer this question, please click the link to protocols.io in the Materials and Methods section of the manuscript (if a link has been provided) or consult the step-by-step protocol in the Supporting Information files.

The step-by-step protocol should contain sufficient detail for another researcher to be able to reproduce all experiments and analyses.

Reviewer #1: Partly

Reviewer #2: Yes

3. Does the protocol describe a validated method?

The manuscript must demonstrate that the protocol achieves its intended purpose: either by containing appropriate validation data, or referencing at least one original research article in which the protocol was used to generate data.

Reviewer #1: Yes

Reviewer #2: Yes

4. If the manuscript contains new data, have the authors made this data fully available?

The PLOS Data policy requires authors to make all data underlying the findings described in their manuscript fully available without restriction, with rare exception (please refer to the Data Availability Statement in the manuscript PDF file). The data should be provided as part of the manuscript or its supporting information, or deposited to a public repository. For example, in addition to summary statistics, the data points behind means, medians and variance measures should be available. If there are restrictions on publicly sharing data—e.g. participant privacy or use of data from a third party—those must be specified.

Reviewer #1: Yes

Reviewer #2: Yes

5. Is the article presented in an intelligible fashion and written in standard English?

PLOS ONE does not copyedit accepted manuscripts, so the language in submitted articles must be clear, correct, and unambiguous. Any typographical or grammatical errors should be corrected at revision, so please highlight any specific errors that need correcting in the box below.

Reviewer #1: Yes

Reviewer #2: Yes

6. Review Comments to the Author

Please use the space provided to explain your answers to the questions above. You may also include additional comments for the author, including concerns about dual publication, research ethics, or publication ethics. (Please upload your review as an attachment if it exceeds 20,000 characters)

*Reviewer #1: Comments to the authors*

*This manuscript presents a new Yucatan mini swine model for sustained AFib which overcomes many of the limitations of previous large-animal models by capitalizing on the anatomical and physiological consistency of the Yucatan mini swine with that of humans. The major novelty of the study is that it uses implantable cardiac monitors (Medtronic Reveal LINQ™) and Fitbark 2.0 activity trackers to allow continuous monitoring of both arrhythmia and behavioral changes – a substantial translational value.*

*The Yucatan swine animal model is well-given, providing several advantages over other domestic breeds in regard to size consistency and potential long-term investigations. Furthermore, the fact that individual surgical approaches and device modifications, made more extensive operations or those with adverse anatomical conditions feasible, is indicative of good technical performance.*

*Together, the model provides a reliable and clinically applicable system for chronic AFib investigation and therapy assessment, potentially allowing us to better understand the arrhythmia-associated functional impact.*

We thank the reviewer for his/her positive comments.

*Major Weaknesses*

*1.The authors should report exact quantitative details and statistical analysis for their main results?*

*This manuscript does not yet adequately report statistics on the results (time to AFib induction, variability in ventricular rates, extent of activity reduction) and histological changes observed in trial animals (e.g. fibrosis).*

*I also invite them to add a more complete statistical analysis and the corresponding graphical summaries (e.g., time to AFib induction as well as activity and rate variability, in the form of Kaplan-Meier curves and bar plots with error bars, respectively) to ensure the clarity and strength of your findings.*

As suggested, we have provided the following:

1. The time to AFib induction: 18.3±5.7 days with a Kaplan-Meier curve showing the individual data on time to AFib induction.
2. The variability in ventricular rates: 100-180 bpm is cited in the text (Page 10; Lines 14-15).
3. The extent of activity reduction: (Fig 6)
4. Histological changes observed in trial animals (e.g. fibrosis) with new data on Trichrome (Fig 7).

*2. I'd suggest: They could validate their model more thoroughly on the molecular and histopathological level:*

*The present manuscript yields little evidence for atrial remodeling, fibrosis or gene expression changes which are necessary to support the translational relevance of model to human AFib.*

*Integration of echocardiographic and histological analysis, and molecular markers of structural and electrophysiological remodeling, will reveal the pathophysiological relationships between the model and clinical AFib.*

We thank the reviewer for this critique. As suggested, we added histology that show changes in atrial tissue (Fig 7). Persistent AFib in our model was associated with clear structural remodeling of the atrial myocardium. Quantitative analysis of Masson’s trichrome staining of left and right atrial biopsies demonstrated significant increases in collagen deposition with a corresponding reduction in atrial myocardial tissue, indicating replacement of functional myocardium by fibrotic scar (Fig 7). This pattern of enhanced fibrosis and myocyte loss is consistent with atrial cardiomyopathy observed in patients with longstanding AFib and supports that our swine model faithfully recapitulates the histopathological substrate that sustains and perpetuates persistent atrial fibrillation. This information has been added to the text (Page 12 ;Lines 1-11)

*3. Proper discussion of limitations of the model in the discussion section?*

*The manuscript is too focused on strengths of the model without enough discussion of limitations such as: variable ventricular rates, n=6, early death of one animal, and its lack of relevance to other types of AF such as paroxysmal AF.*

*I suggest a fair and honest account on these challenges and their implications on the robustness and clinical relevance of the model. This makes your results more reliable and easier to interpret.*

As suggested, we have expanded the limitations section of our manuscript.

**Limitations**: Our model exhibits variability in ventricular rates, which is a known characteristic of clinical AFib and is present in all clinical studies of AFib. Having ICMs in the animals showed that once they converted to sinus rhythm, they did not revert to AFib no matter what their ventricular responses were in AFib. We agree that the small number does limit the statistical power of the study, but swine models offer high translational relevance due to cardiac similarity to humans. The low number of animals means that the observed outcomes might not be broadly generalizable, and the model's true range of responses may not be fully captured. The early death of one animal highlights the inherent risks and technical challenges associated with complex large animal studies. We focused on persistent AFib because this is the most prerssing clinical problem in term of finding a curve for this disease. While paroxysmal AFib is also a clinical problem, it is often triggered by ectopic beats originating from the pulmonary veins in a less remodeled atrium, a different pathophysiology from the sustained AFib typically developed in pacing models. Therefore, our findings are likely most relevant to the study of persistent AFib and the associated structural remodeling. These limitations do not invalidate the model but provide necessary context for interpreting the results and guide future research directions with larger sample sizes and potentially hybrid models to address different aspects of AFib pathophysiology. This discussion has been added to the text (Page 16; Lines19-23 cont. Page 17, Lines 1-2)

*lAlthough major historical models are mentioned, the manuscript does not provide an in-depth comparison with the more recent and emerging, including transgenic or optogenetic AFib models.*

We thank the reviewer for this comment. An in-depth review of other models of AFib is beyond the scope of our report. Importantly, our team has recently reviewed large and small animal models of Afib and submitted this for publication in PLOS One (Bravo et al., 2025, see citation below). We cannot include all that information here, the major advantages that our model offers are: 1) Yucatan miniswine are easy to work with and remain a manageable size through longitudinal studies, 2) The use of ICMs provides an easy method to continuously define the animal’s rhythm in real time, 3) The FitBark collars enable continuous activity tracking, providing a surrogate for quality of life and thus clinical relevance.

Here is the citation: Bravo F, Ref J, Riemenschneider J, Lefkowitz E, Mostafizi P, Salciccioli A, DiPonio S, Grijalva A, Benson D, Tulino AS, Pierce MK, Goldman S, Moukabary T. Development of a customizable pacing system to induce persistent atrial fibrillation in swine. PLOS ONE. 2025; Submitted.

There is recent work on including transgenic or optogenetic AFib models. Importantly, this work has been done mice, rats, and in isolated atrial tissue, where investigators have developed new ways to study the mechanisms of AFib and potentially can be applied to study control electrical activity the role of rotors (Nakato 2023, Nyns 2023). These approaches will potentially help develop new therapies based on these platforms, but they ultimately will have to be investigated in large animal models of persistent AFib. Our model provides an ideal prototype where investigators can define the precise onset of AFib, how long it lasts and its effects on clinical activity. Lastly, tissue from our model will provide gene and protein analyses in a clinically relevant model. This discussion has been added to the text (Page 15, Lines 4-12)

Nakao M, Watanabe M, Miquerol L, Natsui H, Koizumi T, Kadosaka T, Koya T, Hagiwara H, Kamada R, Temma T, de Vries AAF, Anzai T. Optogenetic termination of atrial tachyarrhythmias by brief pulsed light stimulation. J Mol Cell Cardiol. 2023 May;178:9-21. doi: 10.1016/j.yjmcc.2023.03.006. Epub 2023 Mar 24. PMID: 36965700.

Nyns ECA, Portero V, Deng S, Jin T, Harlaar N, Bart CI, van Brakel TJ, Palmen M, Hjortnaes J, Ramkisoensing AA, Zhang GQ, Poelma RH, Ördög B, de Vries AAF, Pijnappels DA. Light transmittance in human atrial tissue and transthoracic illumination in rats support translatability of optogenetic cardioversion of atrial fibrillation. J Intern Med. 2023 Sep;294(3):347-357. doi: 10.1111/joim.13654. Epub 2023 Jun 21. PMID: 37340835.

*Would like to see more of comparative analysis between your model and current large animal models and incorporate knowledge of modern techniques. This should help place your work more effectively in the changing AFib research environment.*

We agree with the reviewer and have added a response in our comments above.

*Minor Points for Revision*

*•Grammar and Style: Clarify sentences and eliminate slangy language (e.g., "pigs presumably feel better").*

We have attempted to remove all “slangy language,” pigs presumably feel better" has been deleted.

*•Figures: 1) Other than captions figure quality and resolution is needed (e.g., Figure 3 needs statistics and clearly labeled axes).*

We added captions to all the figures and statistical data has been added to our new Fig 6.

*•Data Availability: raw ECG and summary data should be made available to the research community in an appropriate format.*

We have made raw ECG and summary data available as suggested.

*Reviewer #2: This paper compares previous AFib models with swine and focusing on key methodological differences as well as outcomes. The manuscript is organized nicely. Following comment would strengthen manuscript.*

*Comment 1: In the result section*

*•It is not clear in terms of clarity and conciseness, e.g. Persistent AFib was induced in ⅚ subjects within 60 days. It should be 5/6 along with %*

This has been corrected in the revised test. “Atrial fibrillation was successfully induced in 4 of 6 animals within 80.3±22.3 days of initiating pacing with 3 animals going into persistent AFib and one animal going into paroxysmal AFib. (Page 10 ; Lines 7-19 ).

*•In addition, reorganization contents would help.*

*•It is unclear about cited reference “Pharmacologic intervention with beta-blockers and calcium channel blockers did not control rapid ventricular rates in one subject (Bishop & Akram, 2021; Pratt et al., 1983)”*

These articles provide clinical context illustrating how beta-adrenergic blockade and diltiazem are used to control ventricular responses in patients premature ventricular beats and with AFib.

*•A separate Protocol adjustments section is highly recommended along with reason of amendment.*

This has been added to the text as suggested (Page 10; Lines 21-22 cont. Page 11, Lines 1-14).

*•A tabular comparison which includes animal model, clinical limitation and clinical relevance would add more weightage.*

The reviewer requests that “*A tabular comparison which includes animal model, clinical limitation and clinical relevance would add more weightage*.” We agree with the reviewer our team has recently reviewed animal models of large and small animal models of Afib and submitted this to PLOS One (Bravo et al,. 2025). This article includes a tabular comparison as suggested by the reviewer. As noted above in response to Reviewer No. 1, we cannot include all that information in this report but our model offers advantages over those previously developed: 1) Yucatan miniswine are easy to work with and remain a manageable size through longitudinal studies, 2) The use of ICMs provides an easy method to continuously define the animal’s rhythm in real time, 3) The FitBark collars enable continuous activity tracking, providing a surrogate for quality of life and thus clinical relevance. This information has been added to the text (Page 13 ; Lines 21-22 cont. Page 14, Lines 1-5).

Here is the citation: Bravo F, Ref J, Riemenschneider J, Lefkowitz E, Mostafizi P, Salciccioli A, DiPonio S, Grijalva A, Benson D, Tulino AS, Pierce MK, Goldman S, Moukabary T. Development of a customizable pacing system to induce persistent atrial fibrillation in swine. PLOS ONE. 2025; Submitted. https://doi.org/10.64898/2025.12.01.691684.

*7. PLOS authors have the option to publish the peer review history of their article (what does this mean?). If published, this will include your full peer review and any attached files.*

If you choose “no”, your identity will remain anonymous but your review may still be made public.

Do you want your identity to be public for this peer review? For information about this choice, including consent withdrawal, please see our Privacy Policy.

Reviewer #1: No

Reviewer #2: Yes: Prakash Sojitra, PhD

We thank you for your review and look forward to your feedback. We believe this manuscript has improved a great deal. Thank you!

Sincerely,

Steven Goldman MD

C. Leonard Pfeiffer Endowed Professor Cardiovascular Medicine

University of Arizona

Sarver Heart Center

PO Box 245046

Tucson, AZ 85724
