# Supplementary material for: Yucatan Miniswine Model of Atrial Fibrillation: Clinical Relevance
Source: PLoS One. 2026 Feb 4;21(2):e0340985. doi: 10.1371/journal.pone.0340985 (PMC12871992; doi:10.1371/journal.pone.0340985)
Supplement: Attachment — Submitted filename: Reviewer Attachement.docx [file pone.0340985.s002.docx]

Dear Editor,

I would like to emphasize that this manuscript describes a rigorously developed and clinically relevant large-animal protocol for inducing persistent atrial fibrillation, addressing several well-recognized limitations of existing models. The protocol demonstrates reproducibility, long-term survivability, continuous rhythm monitoring, and functional assessment, thereby offering clear translational value. Importantly, it provides detailed technical solutions that enable other laboratories to successfully replicate a persistent AF model in a clinically meaningful setting. We believe these strengths make the protocol a valuable methodological contribution to the field, independent of downstream mechanistic or interventional studies.

Kind regards,
Mitko Mladenov
